# Differences of Longitudinal Chromatic Aberration (LCA) between Eyes with Intraocular Lenses from Different Manufacturers

**DOI:** 10.1371/journal.pone.0156227

**Published:** 2016-06-03

**Authors:** Masashi Nakajima, Takahiro Hiraoka, Toshiya Yamamoto, Seiu Takagi, Yoko Hirohara, Tetsuro Oshika, Toshifumi Mihashi

**Affiliations:** 1 Department of Information Processing, Tokyo Institute of Technology, Kanagawa, Japan; 2 Development Engineering Dept., Eye Care Company, TOPCON, Tokyo, Japan; 3 Department of Ophthalmology, Institute of Clinical Medicine, University of Tsukuba, Ibaraki, Japan; 4 Department of Ophthalmology, Ushiku Aiwa General Hospital, Ibaraki, Japan; Rush University Medical Center, UNITED STATES

## Abstract

Several researchers have studied the longitudinal chromatic aberration (LCA) of eyes implanted with an intraocular lens (IOL). We investigated the LCA of eyes implanted with yellow-colored IOLs from three different manufacturers: Alcon Inc., HOYA Corp., and AMO Inc. The number of subjects was 11, 16, and 16, respectively. The LCA of eyes implanted with SN60WF and SN60AT (Alcon Inc.), and with XY-1 (HOYA Corp.), was the same as that of phakic eyes. The LCA of eyes with ZCB00V (AMO Inc.) was smaller than that of phakic eyes. The LCA of eyes implanted with Alcon’s and HOYA’s IOLs, but not the LCA of eyes implanted with AMO’s IOLs, was positively correlated with the powers of the IOLs. We also performed simulations to verify the impacts of LCA on visual performance for 4-mm pupil diameter; the simulations were a polychromatic modulation transfer function (MTF) and a visual Strehl ratio computed on the basis of an optical transfer function (VSOTF). We concluded that the differences between the LCA of different manufacturers do not affect visual performances when some extent of higher-order aberration (HOA) exists. The smaller HOA of AMO IOLs may enhance visual performance.

## Introduction

Retinal images are deteriorated by ocular aberrations, including chromatic aberrations. Aberrations are divided into two categories: chromatic aberrations (both longitudinal and transverse) and monochromatic aberrations. Many researchers have studied how chromatic aberrations affect the quality of the retinal image. Ravikumar et al. [1] and He et al. [2] reported that longitudinal chromatic aberrations (LCA) caused greater deterioration of the retinal image than transverse chromatic aberrations (TCA) did. For phakic eyes, both objective and subjective measurements of LCA have been performed by many researchers [3–15] along a wide spectral range, from 400 nm to 1060 nm. Moreover, Artal [16] integrated the diffractive optics that corrects the typical LCA of the human eye into the adaptive optical system, and reported the influences of LCA on visual performance by measuring visual acuity (VA) and contrast sensitivity (CS). The results indicated that VA was improved by correcting either spherical aberration (SA) alone or both SA and LCA, whereas CS was improved only by correcting both SA and LCA.

Chromatic dispersions for IOL materials were investigated in vitro. Since these materials need to be flexible in order to be implanted through a small incision, and must be biocompatible and stable after being implanted, the base materials used are PMMA, silicone, and acrylic, to which several compounds are added to improve these properties. Currently, the materials are being doped by UV-blocking or blue-light-blocking compounds in order to shield the eye from shorter-wavelength light. Siedlecki et al. [17] investigated the chromatic dispersions of IOLs made of PMMA or acrylic. They measured the focal lengths of the IOLs at the wavelengths between 441 nm and 680 nm and assumed that the shape of the IOL as it is has a power in the eye appropriate to the wavelength of 560 nm. Using the assumed shapes, the measured focal lengths of the IOLs, and the refractive indices of aqueous and vitreous humor from Le Grand schematic eye [18], they determined the chromatic dispersions of the refractive index of the IOLs. They reported that the chromatic dispersion of the refractive index changed greatly when base materials were doped by UV-blocking compounds. The Abbe’s number of a single-material acrylic IOL was 52.80, but the Abbe’s numbers of doped-acrylic IOLs were 27.30 for Alcon’s acrylic IOL (SA60AT) or 32.05 for AMO’s acrylic IOL (Tecnis Z9003). Zhao et al. [19] measured the refractive indices and the Abbe’s numbers of acrylic and silicone IOLs by using an Abbe refractometer. The refractive index and the Abbe’s numbers in the results were 1.55 and 37 for Alcon’s acrylic (SA60AT, SN60AT), 1.47 and 55 for AMO’s acrylic (ZA9003), 1.46 and 42 for AMO’s silicon (Z9002), and 1.51 and 43 for HOYA’s acrylic (YA60BB). Their results suggested that chromatic dispersion varied greatly among the manufacturers even when base materials were the same.

LCAs of IOLs were also investigated in vivo. Nagata et al. [20] measured the LCA for 48 eyes with PMMA IOLs and for 49 eyes with acrylic IOLs between 500 nm and 640 nm by using a modified chromoretinoscopy [21]. This chromoretinoscopy is based on the principle of the retinoscope, by means of which the observer measures the refraction of several wavelengths using a phoropter. They reported that the LCA was 0.75 ± 0.18 D for PMMA eyes and 1.20 ± 0.22 D for acrylic eyes. Siedlecki et al. [22] measured the LCA for 14 eyes of acrylic color-type-clear IOLs (SA60AT) and nine eyes of acrylic color-type-yellow IOLs (SN60WF) between 470 nm and 660 nm by using a visual refractometer PR50 (Rodenstock GmbH); the observer seeks the best image point. They reported that the LCA was 1.45 ± 0.42 D for SA60AT, which was significantly larger than that for the phakic eyes; and that the LCA was 1.17 ± 0.52 D for SN60WF, which was not significantly different from that for the phakic eyes. The results of Siedlecki et al. [22] differ from those of Zhao et al. [19] with respect to whether they treat color-type-clear IOLs and color-type-yellow IOLs as identical. Pérez-Merino et al. [23] measured the LCA for nine eyes of acrylic color-type-clear IOL (ZB99, Abbott Medical Optics Inc.) and for nine eyes of acrylic color-type-yellow IOL (SN60WF, Alcon Inc.) by using laser ray tracing (LRT) the measurement wavelengths of which were 532 nm and 785 nm. They expanded the wavefront aberrations using Zernike polynomials and calculated the LCA from the difference between two defocus terms of two wavelengths. They reported that the LCA of ZB99 was 0.46 ± 0.15 D, which was significantly smaller than that of the phakic eyes. The LCA of SN60WF was 0.75 ± 0.12 D, which was not significantly different from that of the phakic eyes.

Optical simulations to evaluate visual performance have been studied by many researchers. Thibos et al. [24] evaluated the methods to calculate the objective refraction manifested by objective wavefront aberrations. They introduced a visual Strehl ratio based on optical transfer function (VSOTF) taking into account the contrast-sensitivity function (CSF) of the eye [25].

In our research for this paper, we clinically measured the LCA and higher-order aberrations (HOAs) of eyes implanted with IOLs from three manufacturers by using a Hartmann-Shack wavefront aberrometer (HSWA) with three different light sources [26]. To our knowledge, this is the first report on the LCA of IOL-implanted eyes measured in vivo using an HSWA. The aim of this study was to confirm the differences between the LCA of eyes with IOLs from different manufacturers and those of phakic eyes, and to confirm the correlation between LCA and a power of IOL. We also estimated the chromatic dispersions of refractive indices of IOLs on the basis of in vitro measurements using an artificial eye. The impacts of LCA on visual performance under real conditions of HOA were investigated using optical simulations.

## Methods

### Clinical measurements

#### Apparatus (HSWA)

We developed an HSWA with three different light sources to measure chromatic aberration. The basis of the HSWA was a near-infrared (NIR) monochromatic HSWA [27, 28], and was the same as that used in our previous study [26]. The HSWA consisted of a microlens array and a charge-coupled device (CCD). The anterior part of the eye was observed during the wavefront sensing using 950-nm NIR light with the other CCD. The same fixation target as that of a commercially available auto-refractometer (RM-8000, Topcon, Japan) was used. The HSWA was originally designed for NIR wavefront sensing in 840 nm with a super-luminescent diode (SLD) as a light source. Our apparatus had two other light sources, with wavelengths of 561 nm and 690 nm. One of these wavelengths, 561 nm, is near the peak of the spectral sensitivity of the human eye. The other, 690 nm, is roughly the midpoint between 561 and 840 nm. Three optical fibers were bundled and closely aligned with each other. The end of each fiber was always placed in conjugation with the fovea of the eye. The fibers, fixation target, and wavefront sensor were moved to compensate for the spherical error of each subject. The details of the apparatus were reported in our previous paper [26].

The refraction and zero-chromatic aberration of the apparatus were calibrated using a reflective artificial eye. Since the artificial eye consists of an off-axis hyperbolic mirror and diffuser, this design provided not only a chromatic aberration−free optics but also an almost totally aberration-free optics for between −9 and +1 D (the RMS is less than λ/14). Objective refraction and HOA measured in 840 nm were calibrated. The details of the artificial eye are also reported in our previous paper [26].

#### Subjects

We measured 43 eyes of 36 subjects implanted with IOLs who were without cycloplegia. Although two of the subjects had suffered preretinal bleeding or vitreous hemorrhage before their cataract surgeries, none of the participants exhibited retinal abnormalities when their eyes were measured after the surgeries. Average and standard deviations (SD) of spherical equivalents (SE) were −0.91 ± 1.73 D (range: from −7.88 to +1.00), and the ages of the subjects were 70.8 ± 9.5 years (range: 45–87). The powers of the IOLs were 19.6 ± 4.6 D (range: 7–26). Types of IOL implanted in the eyes of the subjects were in the following proportion: four eyes of four subjects had the SN60WF (Alcon Inc.); seven eyes of five subjects had the SN60AT (Alcon Inc.); five eyes of four subjects had the NY-60 (HOYA Corp.); 11 eyes of nine subjects had the XY-1 (HOYA Corp.); and 16 eyes of 14 subjects had the ZCB00V (Abbott Medical Optics Inc.). The primary material of each IOL was acrylic, and each was doped with several compounds for absorbing blue light. The SN60WF and SN60AT IOLs are similar except that the posterior surface of the former is aspheric, whereas the posterior surface of the latter is spherical. In consequence, these IOLs induced different degrees of spherical aberration (SA) and different optimal focal points. But given our conditions of measurement and analysis—with the pupil diameter being 4 mm and our interest being in the difference between the focal points of different wavelengths—we could ignore this difference in SA in the present study. We calculated the differences in optimal focal points between 561 nm and 840 nm regardless of whether the average SA was included. We used the measured average SA of SN60AT, 0.08 μm, in this study. The differences in LCA between 561 and 840 nm were about 0.003 D for both Alcon’s IOL and AMO’s IOL. The impact of the value of SA, 0.08 μm, on LCA was negligible regardless of the kind of IOL being considered.

This study was approved by the Institutional Review Board (IRB) at the Tsukuba University Hospital and adhered to the tenets of the Declaration of Helsinki. Participants provided their written informed consent to participate in this study. The IRB approved this consent procedure.

#### Measurements of human eyes

We consecutively measured ocular wavefront aberrations at the three wavelengths by changing the light source every time we measured an aberration using the HSWA. Each sequence, which consisted of three measurements at three wavelengths, took less than a second. The exposure time was 250 ms for 840 nm, 300 ms for 690 nm, and 300 ms for 561 nm. Because the target was fogged like an auto-refractometer before measurement, the focal point of the subject was the far point in the measurement. The analysis pupil diameter was 4 mm. The details of the measurement protocol were reported in our previous paper [26]. Statistical analyses were performed to draw the correlations between LCA and age or power of IOL. We used statistical software (R version 3.0.2 [29]) for all statistical analyses in this paper.

We also performed a best corrected visual acuity (BCVA) test using a Landolt C chart for the subjects with IOLs. The distance between the subjects and the chart was five meters. Although the test was performed in decimal units of visual acuity, we converted the results to logMAR units.

### Curve fitting and evaluation of visual performance

#### Fitting to Cauchy’s equation of chromatic dispersion

The LCA is defined as the difference between two focuses in two wavelengths; it equals the difference between the SEs of two wavelengths (LCA_λ1-λ2_). To assess chromatic dispersion, we fitted the measured LCA to the chromatic dispersion equation. We used Cauchy’s equation for the chromatic dispersion of refractive index in Eq 1 and for the refraction in Eq 2 that Atchison [30] expanded from Eq 1 to the chromatic dispersion of ocular power. When we fitted with three measurement points, we dropped the order and used Eq 3.

$$n(\lambda )=A+\frac{B}{\lambda^{2}}+\frac{C}{\lambda^{4}}+\frac{D}{\lambda^{6}},$$ (1)

$$SE(\lambda )=A+\frac{B}{\lambda^{2}}+\frac{C}{\lambda^{4}}+\frac{D}{\lambda^{6}},$$ (2)

$$SE(\lambda )=A+\frac{B}{\lambda^{2}}+\frac{C}{\lambda^{4}},$$ (3)

where A, B, C, and D are parameters determined by the measured data. We selected Cauchy’s equation because it was reported by Atchison to be a useful guide up to at least 900 nm. Cornu’s equation is also reported to be useful, but there was not much difference between the two equations for visual simulations.

#### Visual performance

A pupil function, *H*_*λ*_*(x*,*y)*, is defined in Eq 4 with a wavefront aberration, *W*_*λ*_*(x*,*y)*. We expanded the wavefront aberrations to the Zernike polynomials and applied the chromatic dispersion of ocular power offset at 555 nm to Zernike’s defocus term to derive the pupil functions for each wavelength.

$$H_{\lambda }(x,y)=\exp(ikW_{\lambda }(x,y)),$$ (4)

where *k* is wavenumber. The OTF can be calculated as an autocorrelation of a pupil function or by a double Fourier transform method. We can calculate the polychromatic OTF by taking the sum of OTFs for wavelengths. When we consider the photopic luminous efficiency function (LEF) [31], which is regulated by the International Commission on Illumination, V(λ), we can calculate the polychromatic OTF by applying LEF as a weighted-function, as shown in Eq 5. When we do not consider LEF, LEF is set to one regardless of wavelengths. The modulation transfer function (MTF) is defined as an absolute value of OTF as shown in Eq 6.

$$OTF(r,s)=\frac{\int V(\lambda )\cdot OTF_{\lambda }(r,s)d\lambda }{\int V(\lambda )d\lambda },$$ (5)

MTF(r,s)=|OTF(r,s)|, (6)

where *r* and *s* indicate the coordinates in the spatial frequency domain corresponding to the coordinates x and y in the real space.

The formula for calculating VSOTF is described in the previous paper [24]. For the sake of simplicity, the cutoff frequency was a constant regardless of wavelengths, and we calculated the VSOTFs in the range from −60 to +60 line pairs per millimeter, as Ravikumar et al. did [1]. In addition, we took the sum of real components and imaginary components after components had been integrated independently, as Thibos et al. did [24].

#### Conditions for visual simulations

In this section, we explained two MTF calculations with different conditions. Firstly, to investigate the impacts of LCA on visual performance, we calculated the MTF for IOL eyes with LCA but without HOA. That is, when we calculated a pupil function, only the defocus term of Zernike polynomials was set to an adequate value according to the LCA of IOLs for wavelengths. Secondly, to investigate the impacts of HOA on visual performance, simulations were conducted for phakic eyes with LCA and with HOA. We assumed astigmatism can be corrected by the spectacles and astigmatism was excluded from our simulations.

We also investigated the impacts of HOA on VSOTF. The ranges of HOA RMS of our HSWA measurements were 0.07–0.31 um and 0.07–0.46 um for phakic eyes and eyes with IOLs, respectively (HSWA measurements for phakic eyes were performed in our previous study [26]). We picked up subjects for the phakic eyes whose ID numbers were 12, 23, and 27, and whose total HOA RMS were 0.07, 0.16, and 0.27 um, respectively. Given our previous results, which showed an average HOA RMS for phakic eyes of 0.15 μm, the HOA of the subject with ID 23 was typical. We also picked up each two subjects for each manufacturer. The ID numbers were 104 and 106 for Alcon, 127 and 118 for HOYA, and 129 and 140 for AMO. Total HOA RMSs were 0.07 and 0.28 um for Alcon, 0.16 and 0.30 um for HOYA, and 0.14 and 0.32 um for AMO. For those subjects, we calculated VSOTFs with HOA alone, that with LCA alone, and that with both HOA and LCA.

VSOTFs were also calculated for all phakic eyes and eyes with IOLs when both their HOA and LCA were considered. When we calculated the VSOTF, we calculated through-focus VSOTF with the image distances ranged from −0.5 mm to +0.5 mm, and determined the local maximum. We used the local maxima for the analysis.

All simulations for the phakic eyes and for the eyes with IOL included the LEF, and were performed with the image distances set to the equivalent focal length of the typical eye, 17 mm. We applied the same HOA of 840 nm to all wavelengths. The pupil diameter was 4 mm and the spectral range was between 480 nm and 630 nm; the spectral range thus included extrapolation from our measured data. Although it would have been better to use the spectral region between 400 nm and 700 nm for the vision, we would have needed to estimate the chromatic dispersion using the results at three wavelengths (561 nm, 690 nm, and 840 nm) of our instrument for the wavelength region shorter than 561 nm. Because the chromatic dispersion in the shorter wavelength region was an extrapolation, the result might have been ambiguous. So, we restricted the spectral region to 480 nm as the edge of the shortest wavelength for the visual simulation and to 630 nm as the edge of the longest wavelength, on the assumption that a symmetrical region with respect to the peak of spectral sensitivity, 555 nm, was better.

### Modeling the chromatic dispersion

#### Refractive index measurements of IOLs with spectral reflectivity

We measured the spectral reflectivity of the IOLs of each manufacturer in order to derive the chromatic dispersion of refractive index of the IOLs by using a micro-spectrometer (USPM-RU-W, Olympus Corp.), which measures Fresnel reflectivity. We measured the SN60WC (Alcon Inc.), the XY-1 (HOYA Corp.), and the ZCB00V (Abbott Medical Optics Inc.). The XY-1 and ZCB00V IOLs were the same kinds as those implanted in the subjects in the clinical measurements. The only difference between the SN60WC and SN60WF implanted in subjects in clinical measurements is whether the aspheric surface is on the anterior surface or the posterior surface; so we concluded that the chromatic dispersion properties of these two types of IOL were similar. The powers of the IOLs were +10, +20, and +30 D. We used a 40x objective lens to keep reflection at the posterior surface from entering the detector. We used optical glass S-TIH4 or S-BSL7 (OHARA Corp.) as references. The refractive indices of IOLs were calculated on the basis of the reflectivities of IOLs; the manufacturing error of refractive index of the reference glass for d-line is ± 0.0003 and is sufficient for the references. We performed the measurements only when the surfaces of the IOLs were dry and the temperature was 25°C.

#### IOL shapes with interferometry

We also measured the anterior and posterior curvatures and the thicknesses of the nine IOLs by using an interferometer (Verifire^TM^ XPZ, Zygo Corp.). The interferometer was based on phase-shift interferometry; the method we used to measure the curvature was described in detail by Selberg [32]. The light source was a built-in 632.8 nm laser. We properly used two reference lenses, the F-numbers of which were 3.3 and 7.2, according to the curvatures of the IOLs. Each measured IOL was held on a stage with a laser displacement gauge that is accurate to within one μm. Because the IOLs are not perfect spheres, we measured the curvature of the center of the IOLs. The optical thickness of the IOL was measured using the distance between two *Cat’s eye* positions [32] on the anterior or posterior surfaces, and the actual thickness of the IOL was measured by multiplying the optical thickness by the refractive index. We can more accurately calculate the actual thicknesses of IOLs when an F-number of reference lens and a refraction on an anterior surface are considered.

#### IOL artificial eye

**Fig 1:**
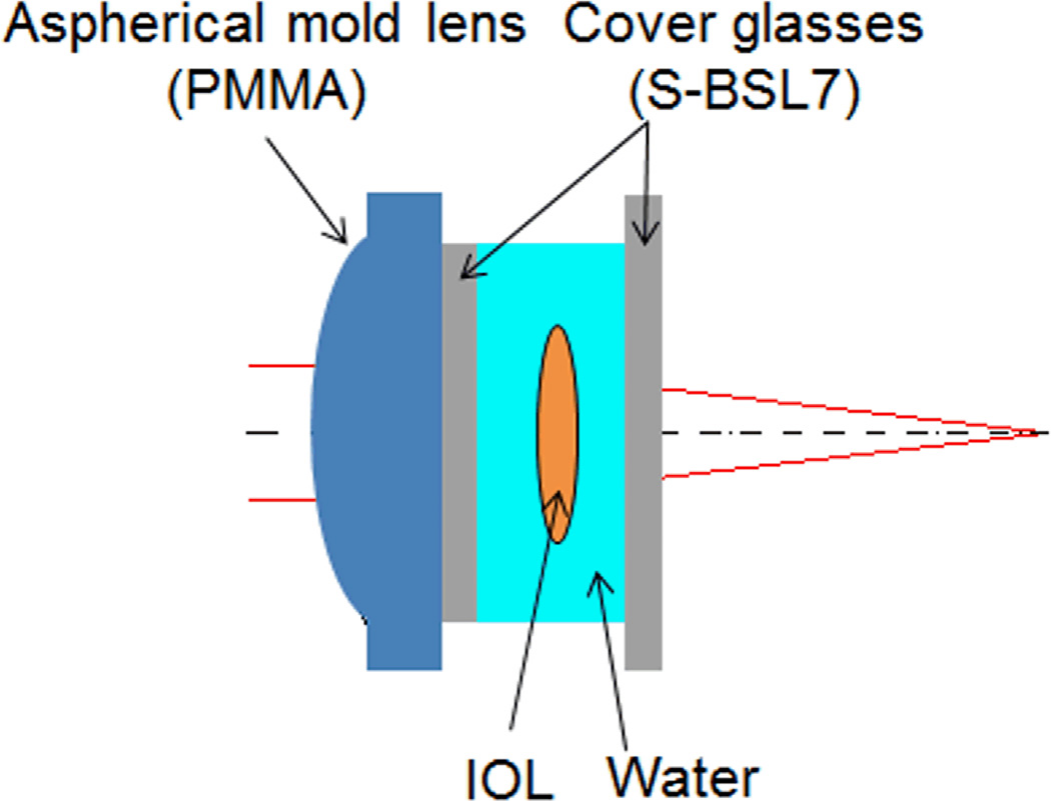
Schema of IOL artificial eye.

We made an artificial eye that can hold an IOL in order to investigate the properties of IOLs. Campbell [33] made an artificial eye in which an IOL can be set. It had unique characteristics: the IOL was optically held in water; the anterior lens, made of polymethyl methacrylate (PMMA), mimicked cornea; and the retinal surface was a scattering surface that could be moved to adjust to the focal point of the artificial eye. The artificial eye that we made, which had the same characteristics as Campbell’s artificial eye, was used to measure the LCAs for the nine IOLs and to evaluate the chromatic dispersions of refractive indices of the IOLs. In addition, the anterior molded lens of our artificial eye was aspheric-designed, so that the SA was equal to zero. The schema of the artificial eye is shown in Fig 1 and the optical lens data are shown in Table 1. We adjusted the retinal length until the measured spherical equivalent was equal to zero. Using a heater, we kept the temperature of the water at 35°C, about the same as human body temperature. We used the refractive index, Abbe’s number, and curvature of PMMA molded lens that are published by the manufacturer.

**Table 1:** Optical lens data of the artificial eye.

Surface No.	Radii [mm]	Thickness [mm]	Material
1	12.4460 (Aspheric)	3.46	PMMA (n = 1.495, ν_d_ = 61.4)
2	∞	1.0	B270 (SCHOTT AG)
3	∞	2.5 ± α*	Water
4	Anterior radii of IOL	b**	IOL
5	Posterior radii of IOL	4.7 ± c*	Water
6	∞	1.0	B270 (SCHOTT AG)
7	∞	free to change	Air

* lengths depend on the radii and thickness of the IOL.

** actual thickness of the IOL.

#### Chromatic dispersion of IOL in an artificial eye

We analyzed the LCA of an eye with an IOL using paraxial ray tracing. The goal of the analysis was to estimate the LCA of the IOL and the chromatic dispersion of IOL materials on the basis of the artificial-eye measurements. The LCA caused by the i-th surface is expressed with the paraxial approximation in Eq 7 [34].

$$\Delta d_{i}=\frac{y_{i}\cdot n_{i-1}\cdot \phi_{i}}{n_{k}u_{k}}\left(\frac{\Delta n_{i-1}}{n_{i-1}}-\frac{\Delta n_{i}}{n_{i}}\right),$$ (7)

where, *y*_*i*_, *n*_*i*_, *ϕ*_*i*_, *u*_*i*_, and *Δn*_*i*_ are an incident height, a refractive index, an incident angle, an outgoing angle along the axis after refraction of the i-th surface, and a difference of refractive indices between two wavelengths, respectively. The subscript, *k*, indicates the number of last surfaces of the optics. Because *Δd*_*i*_ is a difference between two focus points of two wavelengths, the difference between two wavelengths derived from the optics, *Δd*, is equal to *Δd*_*1*_+*Δd*_*2*_+…+*Δd*_*k*_.

Refractive power of the eye in diopter and then LCA in diopter are expressed in Eq 8 and in Eq 9.

$$power=n_{k}\cdot \frac{1000}{f},$$ (8)

$$LCA=n_{k}\cdot \left(\frac{1000}{f+\Delta d}-\frac{1000}{f}\right),$$ (9)

where *f* is a focal length in the last element of the optics in millimeters.

Our artificial eye consisted of three refractive surfaces (Fig 1). The calculated LCA was related to the measured LCA as follows: *Δd*_*i*_ in the left-hand side of Eq 7 was replaced by the measured LCA. And *y*_*i*_, *ϕ*_*i*_, *u*_*i*_, and the known *Δn* in the right-hand side of Eq 7 were replaced by the values calculated by using paraxial ray tracing. Then we obtained the *Δn* of the IOL, which was the only unknown value.

#### LCA paraxial ray traced for the human eye implanted with IOL

With the paraxial approximation, we were able to calculate the differences in refractive index, *Δn*, of IOLs between two wavelengths using Eq 7 and the resulting LCA of HSWA, since the other parameters were known. We obtained the curvature of the anterior surface of the cornea from the keratometer (KR-1, TOPCON, Japan). We estimated posterior curvature of the cornea by applying the specific ratio 6.5/7.8 of the Le Grand Schematic eye [18] to the anterior curvature of the cornea. Dubbelman et al. [35] reported that the ratio between posterior and anterior curvatures of the cornea was approximately constant, 0.83. We used the chromatic dispersions from the Le Grand schematic eye of the cornea, aqueous humor, and vitreous humor. Because we had the anterior chamber depth (ACD) of only eight of 11 eyes with Alcon’s IOL, we used the average ACD, which was 3.32 mm, of the large number of pseudophakic eyes [36] as the ACD of the other eyes as well. Anterior and posterior curvatures of IOLs and the thicknesses of +10, +20, and +30 D IOLs were obtained from the interferometer measurements. The specifications of the IOLs implanted in the subjects were estimated on the basis of those measured data and the power of IOLs.

We solved a simultaneous equation using Cauchy's equation for two combinations—561 nm and 840 nm, 561 nm and 690 nm (or 690 nm and 840 nm)—for the refractive index of IOL. Then, we could find the coefficients B and C of Cauchy's equation.

Finally, we calculated the paraxial LCA of human eyes implanted with IOLs by applying the refractive-index chromatic dispersion of IOLs to the eyes with IOLs.

## Results

### Clinical data

Table 2 shows summaries of the results. The LCA and SE data are averages of the repeated measurements. The results reported in our previous study for LCA in the case of phakic eyes [26] are also shown in Table 2.

**Table 2:** IOL specifications and summaries of data from each manufacturer.

Manu-facturer /Number of eyes	Model	Lens type of IOL	Age [years]	IOL power [D]	LCA_840-561_ [D]	SE [D]
Alcon	SN60WF	Posterior aspheric	69.1±12.4	+18.5±5.0	+0.96±0.11	−1.06±1.32
Inc./	SN60AT	Spheric	(range:	(range:	(range:	(range:
11 eyes			45–83)	+8.5-+26)	+0.79-+1.11)	−4.50-+0.75)
HOYA Corp./5 eyes	NY-60	Aspheric	71.0±5.6 (range: 64–79	+18.3±1.3 (range: +16.5-+19.5)	+0.80±0.06 (range: +0.69-+0.85)	−0.78±1.09 (range: −2.63-+0.25)
HOYA Corp./11 eyes	XY-1	Aspheric	70.2±12.4 (range: 46–87)	+21.6±3.6 (range: +11.5-+24.5)	+1.01±0.09 (range: +0.83-+1.12)	−0.90±2.68 (range: −7.88-+1.00)
AMO Inc./16 eyes	ZCB00V	Anterior aspheric	72.4±6.0 (range: 63–84)	+19.4±5.4 (range: +7-+24.5)	+0.66±0.06 (range: +0.57-+0.81)	−0.86±1.44 (range: −4.13-+0.50)
Phakic/45 eyes [26]	-	-	35.7±11.5 (range: 22–57)	-	+0.96±0.06 (range: +0.86-+1.09)	−3.75±2.21 (range: −8.25-−0.22)

The resulting averages and SDs of the BCVA test in logMAR units were −0.06 ± 0.04 (range: −0.06 - +0.05) for the IOL of Alcon, −0.06 ± 0.04 (range: −0.08–0.0) for the IOL of HOYA NY-60, +0.07 ± 0.24 (range: −0.08 - +0.70) for the IOL of HOYA XY-1, and 0.0 ± 0.08 (range: −0.08 - +0.15) for the IOL of AMO. The differences in BCVA among the IOLs were not statistically significant (ANOVA, p > 0.05).

### Dependency of total LCA on the power of IOL

**Fig 2:**
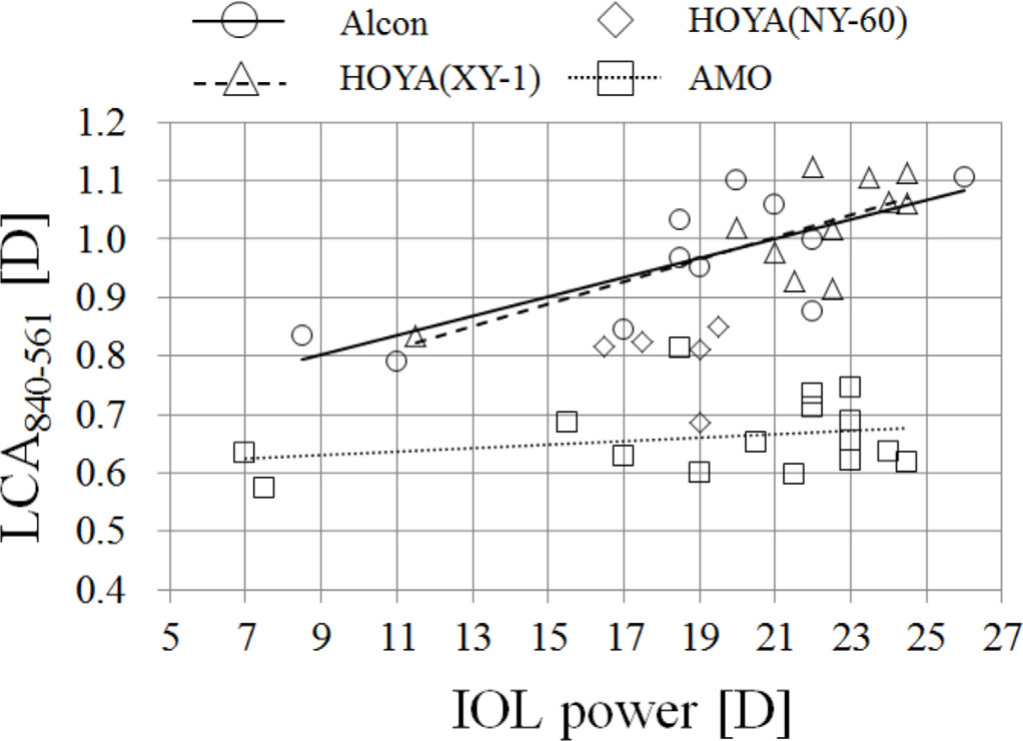
LCA_840-561_ as a function of IOL’s power.

Fig 2 shows LCA_840-561_ as a function of a power of the IOL. The LCAs of IOLs made by AMO were smaller than those of others, suggesting that the IOLs of AMO had smaller chromatic dispersions than the others. The slopes of the regression analysis for IOLs made by Alcon and HOYA XY-1 were statistically significant (p < 0.05). The slope for the IOLs made by AMO was not statistically significant (p > 0.05). We did not perform the regression analysis for the IOLs made by HOYA NY-60 because the data were too few and the power range was too narrow.

### Correlations between LCA and age of subject for the eyes with IOLs

**Fig 3:**
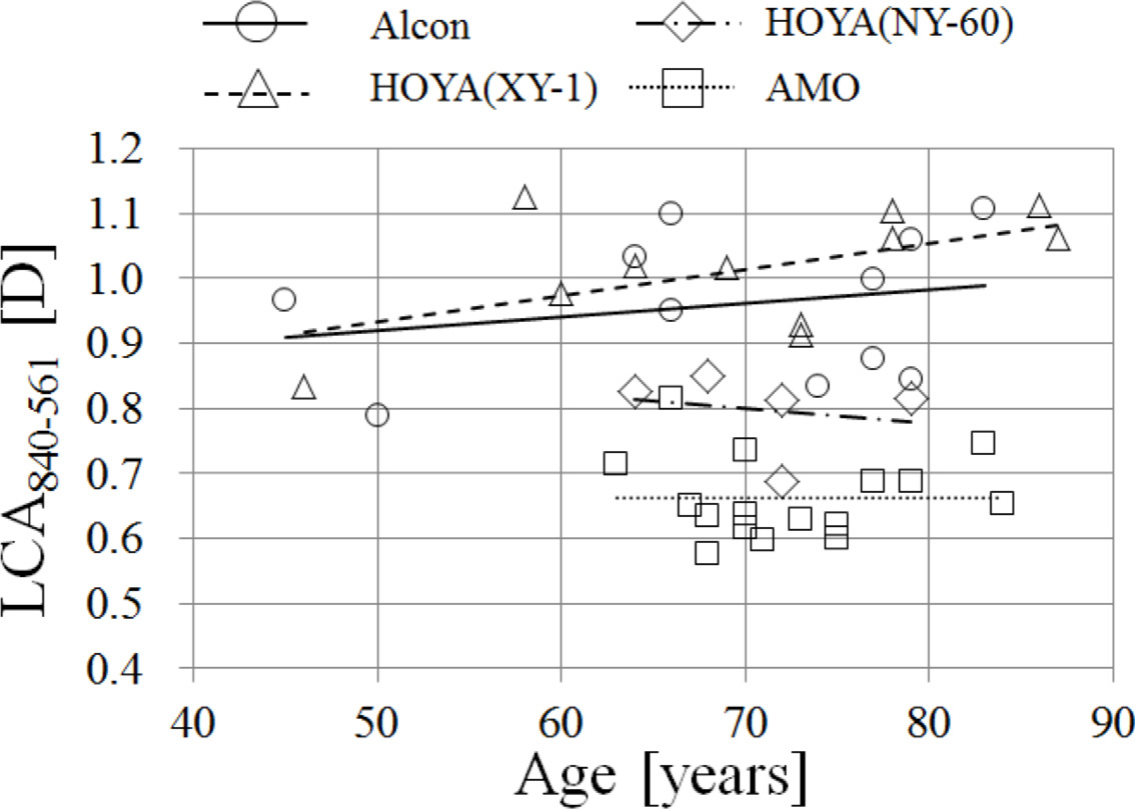
LCA840-561 as a function of age. Dotted lines indicate the prediction intervals, and the dashed line indicates extrapolation of regression beyond the age range.

Fig 3 shows LCA_840-561_ as a function of age. We did not find any age dependency with the LCA_840-561_ of the phakic eyes of subjects between 22 and 57 years old. The regression and prediction intervals for phakic eyes are shown in Fig 3. For all types of IOLs, none of the slopes of regression to age was significant (p > 0.05). The differences in LCA between Alcon, HOYA XY-1, and the phakic eye were not statistically significant; but the differences in LCA were statistically significant for HOYA NY-60 and AMO (ANOVA, p < 0.05; Tukey-Kramer method, p < 0.05). However, because the average power of the IOL of HOYA NY-60 was smaller than that of HOYA XY-1, it was to be expected that HOYA NY-60 would show smaller LCA than that of HOYA XY-1. The LCA of AMO’s IOL between 561 nm and 840 nm had almost 70 percent of the LCA for the phakic eyes.

### Chromatic power dispersion of the eye

**Fig 4:**
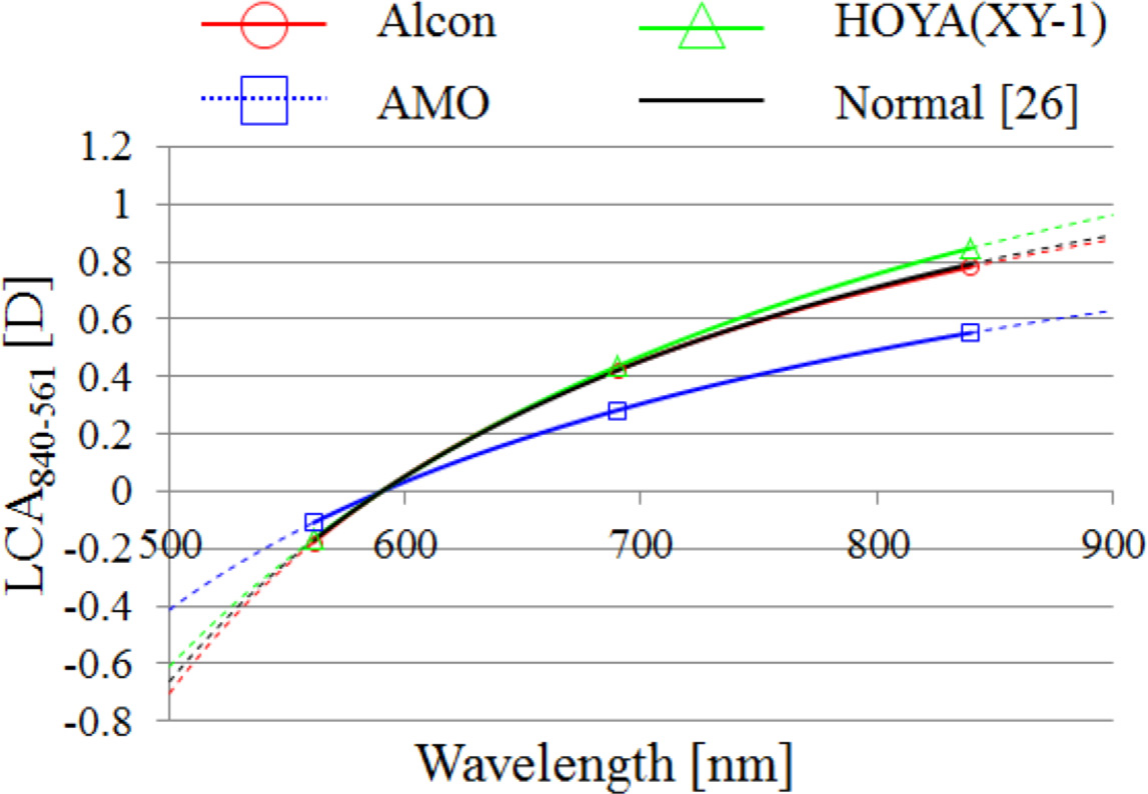
The Cauchyʼs fittings for each average of LCA. Fittings were plotted using the wavelength 590 nm as an offset wavelength. Dotted lines indicate extrapolations of regressions. Symbols indicate measured points for each IOL.

We performed a linear least-squares curve fitting and estimated the power chromatic dispersion in Eq 3 for the IOLs of Alcon, HOYA XY-1, and AMO by using the averages of measurement data of SEs for 840, 690, and 561 nm. The results are shown in Fig 4. We found that the IOLs of Alcon and HOYA XY-1 had LCA similar to that of the phakic eyes, and that the IOLs of AMO had smaller LCA than those of others. The dotted lines indicate extrapolations of regressions beyond the range of our instrument’s spectral region.

### Results of HOA of the eye

**Fig 5:**
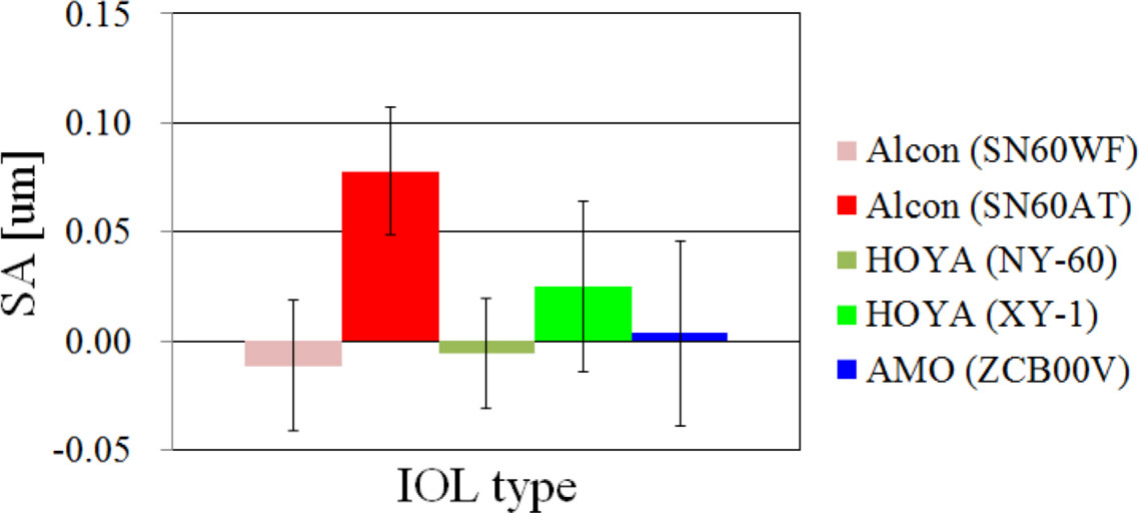
The results of spherical aberration of each IOL type.

**Fig 6:**
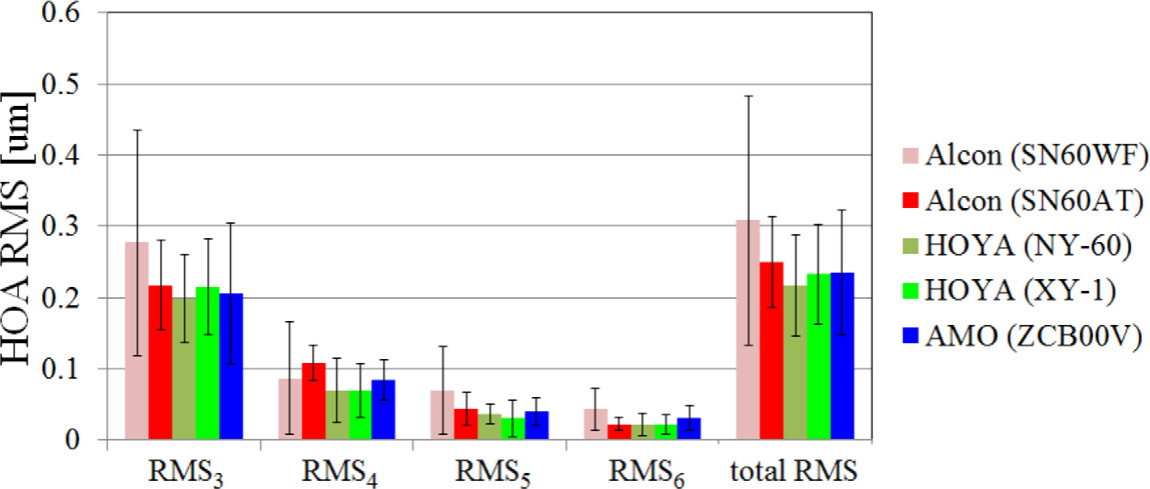
The results of HOA RMSx and total HOA RMS of each IOL type.

The SA of the eye for each IOL type is shown in Fig 5. The results of SA of SN60AT were significantly larger than those of the others (ANOVA; p < 0.05, Tukey-Kramer method; p < 0.05). This made sense because both surfaces of the SN60AT were spherical and at least one surface of the other IOLs was aspheric. The RMS of HOA from 3rd order to 6th order (RMS_3_-RMS_6_) and the total RMS of HOA are shown in Fig 6. The differences in any orders or total RMS were not significant (ANOVA; p > 0.05) even though the SA of the SN60AT was larger than that of the others.

### Influence of the LCA on visual performance

**Fig 7:**
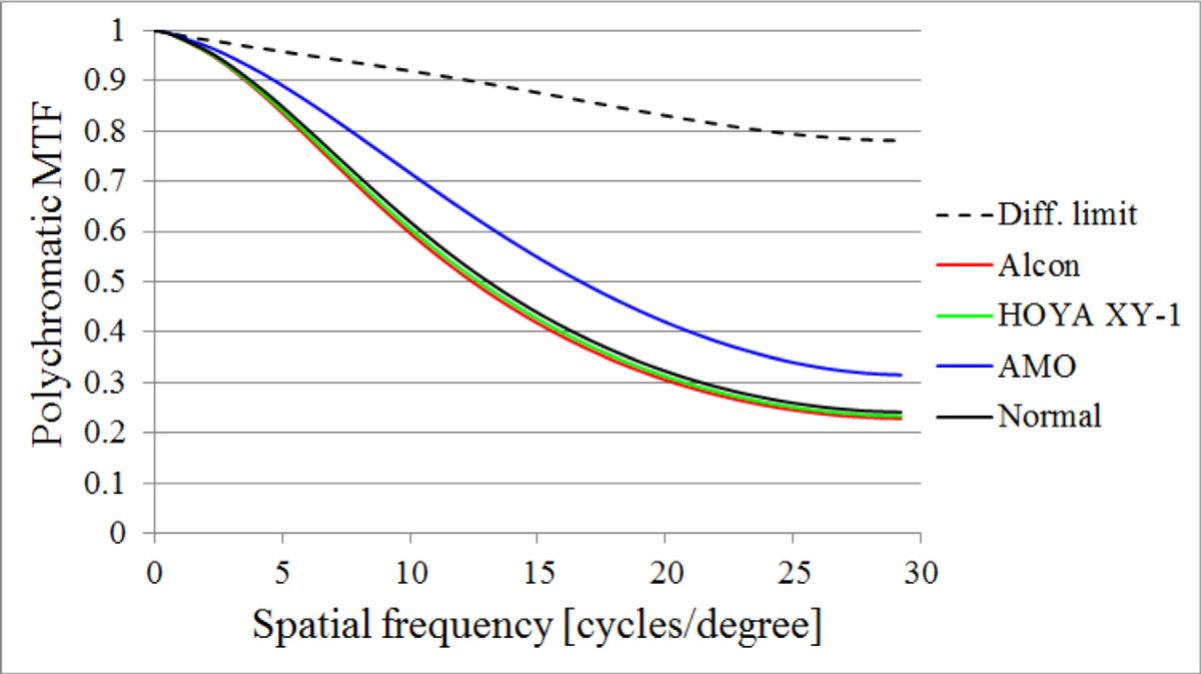
The results of polychromatic MTF simulations with LCA but without HOA. The results of Alcon, HOYA XY-1, and AMO IOLs with the power +20 D, and the average of phakic eyes.

We performed polychromatic MTF simulations with LCA but no HOA in order to investigate the impact of LCA on visual performance. The polychromatic MTFs of the phakic eyes, and of eyes with Alcon, HOYA XY-1, and AMO IOLs, are shown in Fig 7. For the Zernike defocus term, we defined them according to the results of LCA using the wavelength 555 nm as an offset wavelength. The polychromatic MTF of the diffraction limit condition, LCA is always null, is shown in the figure as a dotted line; the pupil function does not include any aberrations, but LEF is considered even though the LCA is null. The LCA for AMO’s IOL showed a relatively higher MTF than that of the others.

### The influence of LCA on visual performance with HOA

#### Polychromatic MTF

**Fig 8:**
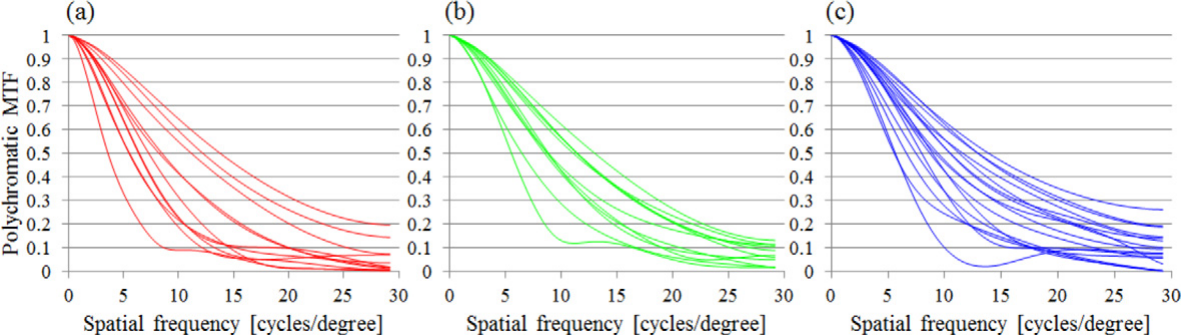
All polychromatic MTF results of IOLs. Alcon (Left), HOYA XY-1 (Center), and AMO (Right) IOLs. Only the MTF in horizontal, with LEF considered, is shown in the figures.

The MTF results for all data of Alcon, HOYA XY-1, and AMO IOLs are shown in Fig 8. We found that the MTFs seemed to vary regardless of IOL type, so that LCA affected imaging performance on the retina to a lesser extent when the IOL was implanted in the eye.

#### VSOTF

**Fig 9:**
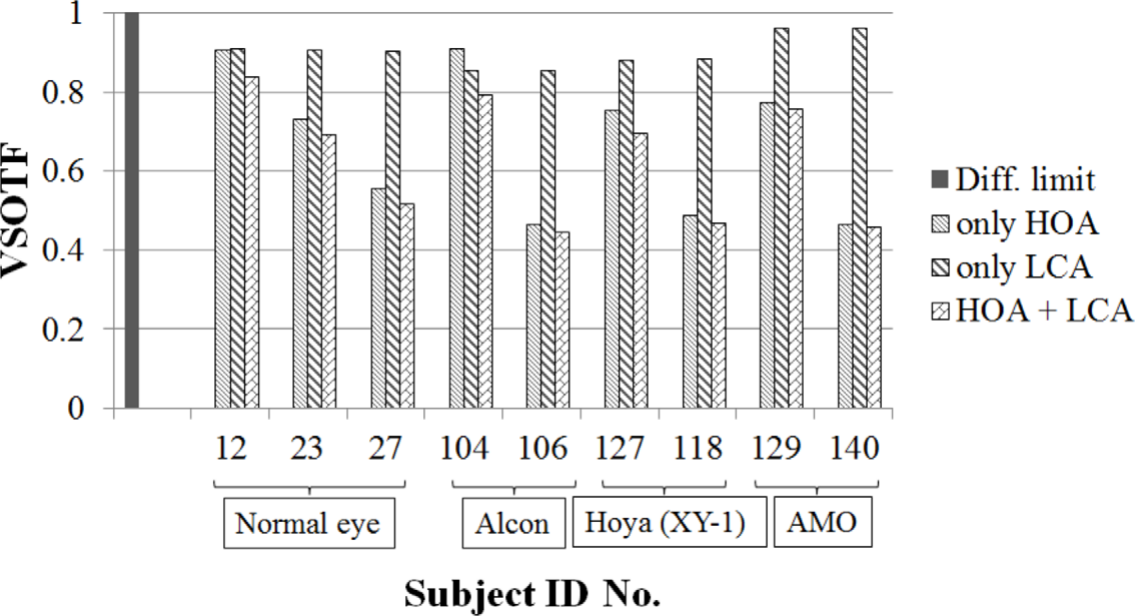
The results of VSOTFs. Results for phakic eyes that have three different degrees of HOA and results for eyes with Alcon’s IOL, HOYA XY-1’s IOL, and AMO’s IOL that have two different degrees of HOA for each manufacturer.

The results of VSOTF for the subjects we picked up are shown in Fig 9. The VSOTFs with HOA alone showed results similar to those with both HOA and LCA in seven subjects, excluding subjects 12 and 104. On the other hand, VSOTFs with LCA alone differed from those with both HOA and LCA.

**Fig 10:**
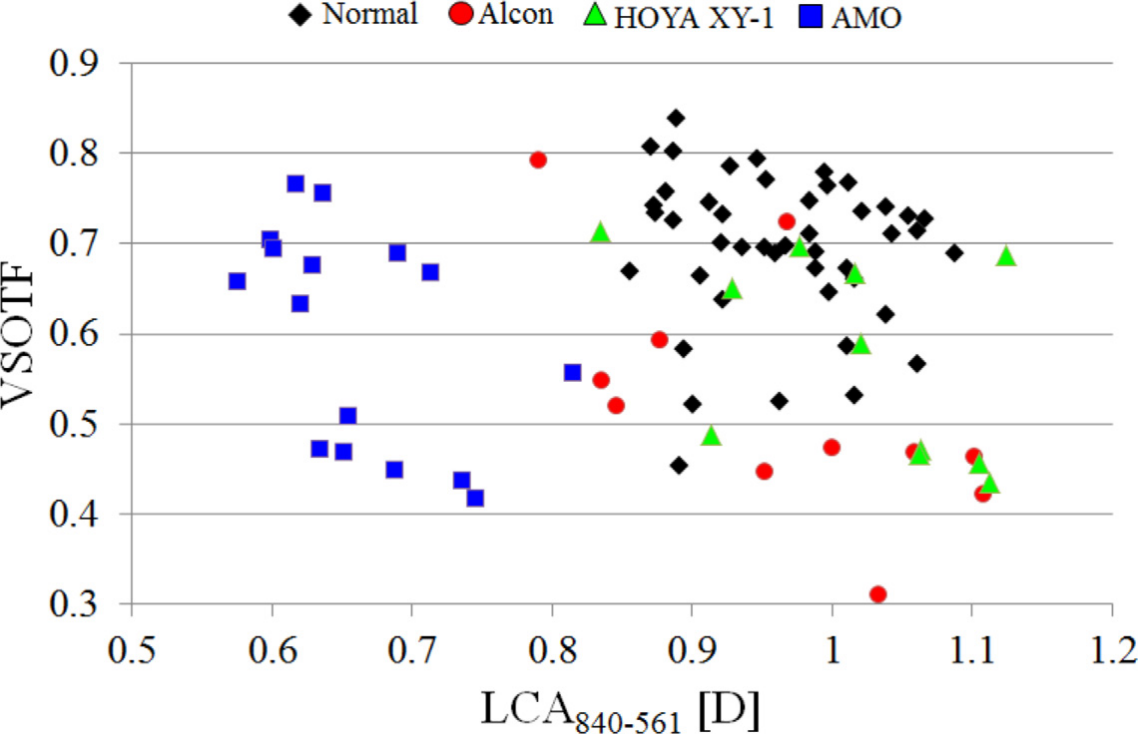
The VSOTFs as a function of LCA840-561. The VSOTFs were calculated by considering both HOA and LCA.

Relationships between VSOTF and LCA are shown in Fig 10. The differences in VSOTF between each manufacturer were not statistically significant (ANOVA, p > 0.05).

**Fig 11:**
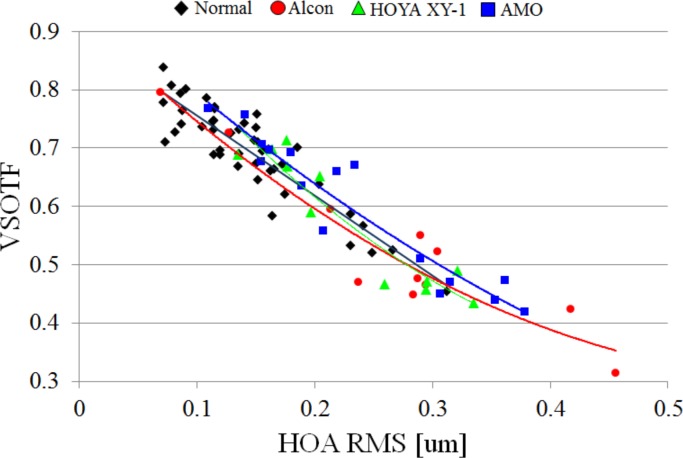
The VSOTFs as a function of HOA RMS. The VSOTFs were calculated by considering both HOA and LCA. The quadratic regressions for each manufacturer and for phakic eyes are also shown.

Relationships between VSOTF and HOA are shown in Fig 11. We found that the VSOTF depended on the HOA regardless of the kind of IOL. The IOLs of AMO that have smaller chromatic dispersion may show relatively high VSOTFs, as indicated by the fact that the regression curve of AMO’s IOL was barely separated from that of others.

### Results of spectral reflectivity and interferometry

The results of reflectivity and interferometry are shown in Table 3. We used only the results of reflectivity of IOLs at 555 nm, because errors of reflectivity in the visible region were small enough to calculate an index; the standard deviation (SD) of the calculated refractive index was less than 0.0002 at 555 nm. In the near-infrared region, the errors of reflectivity were so large that we did not use the results; the SD of the calculated refractive index was about 0.002.

**Table 3:** The results of reflectivity and interferometry. When values from the manufacturers are available, they are provided in parentheses.

Manufacturer	Model name	IOL power [D]	Thickness [mm]	Anterior radius of curvature [mm]	Posterior radius of curvature [mm]	Fresnel reflectivity at 555 nm [%]	Calculated refractive index at 555 nm
		+10	0.378	48.307	43.086		
Alcon Inc.	SN6CWS	+20	0.593 (0.59)	21.677 (21.56)	21.924 (22.00)	4.717±0.0018	1.5549±0.0001 (1.55 at 550 nm)
		+30	0.784	13.364	16.060		
		+10	0.472 (0.50)	33.496 (34.71)	55.793 (51.80)		
HOYA Corp.	XY-1	+20	0.569 (0.58)	17.285 (18.85)	27.392 (23.10)	4.584±0.0029	1.5449±0.0002 (1.548)
		+30	0.796 (0.79)	12.369 (12.95)	16.396 (14.80)		
		+10	0.498 (0.55)	25.946	34.599		
AMO Inc.	ZCB00V	+20	0.704 (0.717)	11.523	17.292	3.710±0.0017	1.4771±0.0001 (1.47)
		+30	0.845 (0.84)	6.350	16.470		

### Results of artificial eye

**Fig 12:**
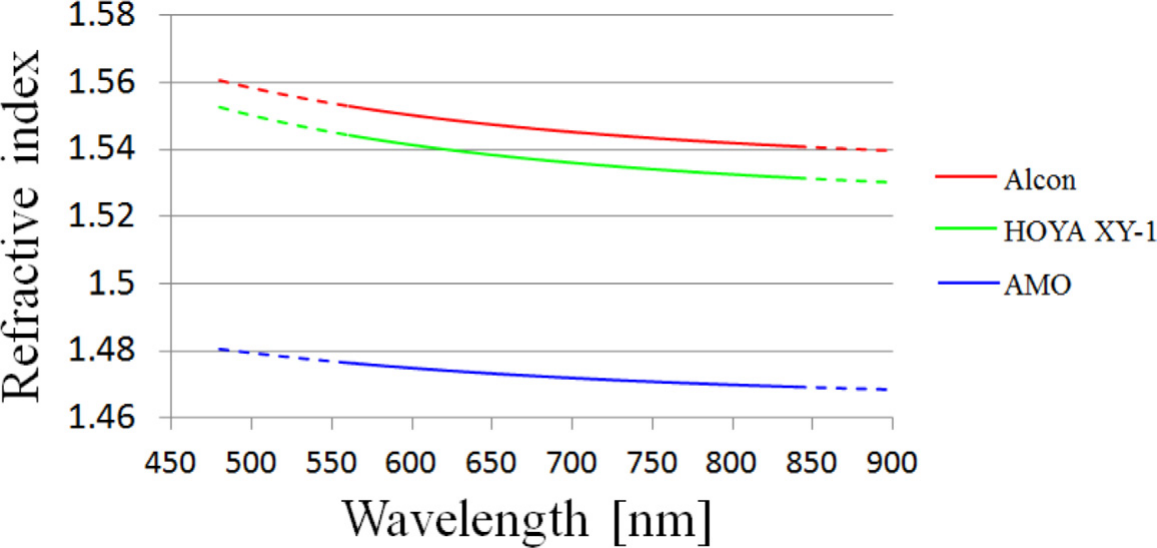
The results of refractive index chromatic dispersion of IOLs. Results correspond to the Cauchy’s equation coefficients in Table 5. Dotted lines indicate extrapolations.

We performed HSWA measurements with an artificial eye in order to estimate the chromatic dispersions of the refractive indices of the IOLs. Table 4 shows the results of LCA_840-561_ and LCA_690-561_ of each IOL. We evaluate the chromatic dispersions of the IOLs on the basis of the HSWA measurements. The temperature of water was maintained at around 35°C and the refractive index of water of a given temperature was calculated by using the equation reported by Schiebener et al. [37]. We used Cauchy’s equation to fit the chromatic dispersion. Table 5 shows Cauchy’s equation coefficients of refractive indices. Table 5 also shows refractive indices at 555 nm or d-line and Abbe’s number calculated by using the Cauchy coefficients. When we performed the fittings to Cauchy’s equation, we used the results of +30 D IOLs, because fitting to large LCA with 30 D artificial eyes provides better accuracy than fitting to small LCA. Fig 12 shows the chromatic dispersion of the refractive index of IOL corresponding to the Cauchy coefficients in Table 5. The dotted lines are extrapolations. The refractive index of AMO’s IOL was relatively small.

**Table 4:** The results of HSWA measurements for the artificial eye.

Manufacturer	Model name	IOL power [D]	LCA_840-561_ [D]	LCA_690-561_ [D]
		+10	0.84	0.49
Alcon Inc.	SN6CWS	+20	0.97	0.55
		+30	1.15	0.73
		+10	0.82	0.49
HOYA Corp.	XY-1	+20	0.97	0.59
		+30	1.14	0.71
		+10	0.58	0.38
AMO Inc.	ZCB00V	+20	0.72	0.45
		+30	0.67	0.43

**Table 5:** Cauchy’s equation coefficients of chromatic dispersion of the refractive index, refractive index of 555 nm and d-line, and Abbe’s number. The chromatic dispersions were estimated by using the HSWA results of IOLs with the power +30 D.

	A	B	C	D	n_555_	n_d_	ν_d_
Alcon	1.53096	7.14599×10^3^	−6.99545×10^7^	-	1.555	1.552	38 (37)
HOYA	1.52122	7.29953×10^3^	−1.15633×10^7^	-	1.545	1.542	39 (36.9)
AMO	1.46273	4.86248×10^3^	−1.72225×10^8^	-	1.477	1.475	67 (55)

### LCA paraxial ray traced and the LCA measured with HSWA for the eye with IOLs

**Fig 13:**
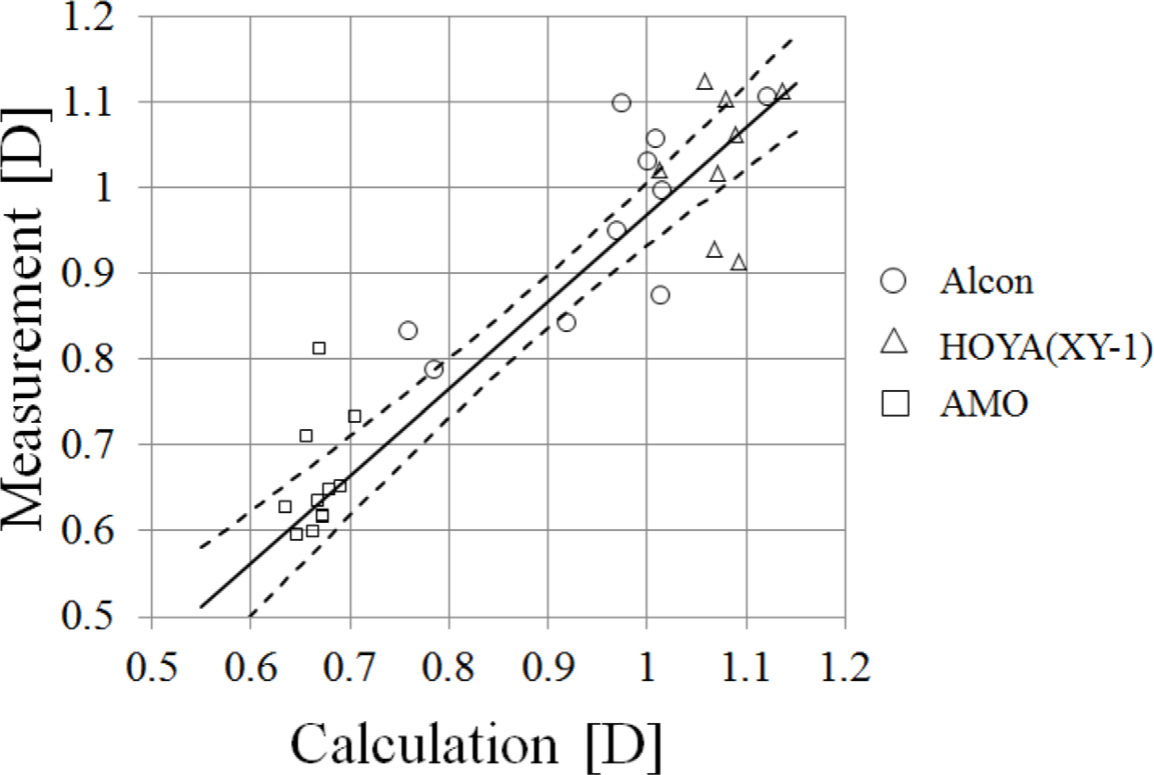
Calculated LCA and measured LCA with HSWA. A Deming regression line is shown as a solid line and confidence intervals (α = 0.05) are shown as dashed lines. We acquired the ACD data for only 8 of 11 of Alcon’s IOLs, and the plot sizes were larger for the results using ACD data.

The relationship between the calculated LCA_840-561_ using Eqs 7–9 and the measured LCA_840-561_ using the HSWA are shown in Fig 13. A Deming regression line [38] based on all of the data is also shown in the figure. The slope and intercept of the regression were 1.02 and −0.05, respectively. The Deming regression is a way to minimize the sum of squares of differences between the regression line and the plot points. The standard deviation (SD) of the differences was 0.04 D for our results. That the differences were not very large suggested that the chromatic dispersions of the cornea, aqueous humor, and vitreous humor that we defined using the Le Grand schematic eye were moderately appropriate, given the chromatic dispersions of the IOLs determined by this study (Table 5).

## Discussion

### Correlations between LCA and age of subject for the eyes with IOLs

Our results indicated that the LCA does not change with age for the eyes with IOLs. Our previous results [26] also indicated that the LCA does not change with age, at least not between the ages of 22 and 57, for the phakic eyes; even though some reports ([6] etc.) indicated that LCA does change with age. The fact that we were able to confirm that LCA does not change with age for the pseudophakic eyes is significant because it suggests that the LCA derived from the cornea alone does not depend on age.

### LCA comparison to previous studies

We compare our LCA results to those of previous studies in Table 6. Nagata et al. [20] reported that the LCA of an acrylic IOL was larger than that of PMMA’s IOL; on the other hand, we found large differences between the LCA of acrylic IOLs. Considering those results, the chromatic dispersion of IOLs with the base material of PMMA was not always smaller than that of acrylic. This study and the study by Pérez-Merino et al. [23] showed that the LCA of AMO’s IOL, which is acrylic, was smaller than that of other manufacturers. Measuring SA60AT and SN60WF from the AcrySof series, Siedlecki et al. [22] reported that the chromatic dispersions of these two types of acrylic IOLs made by Alcon differed significantly even though the powers of the IOLs were approximately the same. The chromatic dispersion of the two types of IOLs may be affected by whether the blue-light-blocking compounds were un-doped or doped. Their reports suggest that the LCA of each IOL may differ at the shorter wavelength when each blue-light-blocking property of the IOLs differs. Our results for Alcon’s IOLs were obtained from the SN60AT and the SN60WF. Both IOLs were yellow-colored because their blue-light-blocking compounds had been doped. The difference between the LCA results for these two types of IOL was not significant (t-test; p > 0.05), and in this study we treated them as belonging to the same group.

**Table 6:** Comparison of our results with other researchers’ results corresponding to comparable spectral regions.

	Nagata et al. [20]	Siedlecki et al. [22]	Pérez-Merino et al. [23]
Methods (apparatus) of other researchers	A modified chromoretinoscopy of Bobier & Sivak	Custom visual refractometer PR50 (Rodenstock GmbH)	Laser ray tracing
Spectral range (nm)	500~640 nm	470~660 nm	532~785 nm
Previous study	Phakic eye (27 eyes) 0.82±0.16 D	Alcon (AcrySof series) (SA60AT) (14 eyes) 1.45±0.42 D	Alcon (AcrySof series) (SN60WF) (9 eyes) 0.75±0.12 D
	PMMA (48 eyes) 0.75±0.18 D	Alcon (AcrySof series) (SN60WF) (9 eyes) 1.17±0.52 D	AMO (ZB99) (9 eyes) 0.46±0.15 D
	Acrylic (AcrySof series) (49 eyes) 1.20±0.22 D		
Our previous study	Phakic eye (45 eyes) 0.84±0.27 D	Phakic eye (45 eyes) 1.19±0.46 D	Phakic eye (45 eyes) 1.04±0.12 D
This study	Alcon (AcrySof series) (SN60WF, SN60AT) (11 eyes) 0.90±0.24 D	Alcon (AcrySof series) (SN60WF, SN60AT) (11 eyes) 1.30±0.40 D	Alcon (AcrySof series) (SN60WF, SN60AT) (11 eyes) 1.06±0.16 D
	HOYA (XY-1) (11 eyes) 0.83±0.22 D	HOYA (XY-1) (11 eyes) 1.16±0.37 D	HOYA (XY-1) (11 eyes) 1.08±0.14 D
	AMO (ZCB00V) (16 eyes) 0.32±0.29 D	AMO (ZCB00V) (16 eyes) 0.45±0.43 D	AMO (ZCB00V) (16 eyes) 0.41±0.36 D

### The relationship between LCA, HOA, and visual performance

The results of Fig 7 indicate that the MTFs of Alcon’s IOL and of HOYA XY-1’s IOL were approximately the same, whereas AMO’s IOL showed a larger MTF than the others did. The results suggest a simple fact: image on the retina improves as the LCA becomes smaller if there are no aberrations but the LCA.

On the other hand, the results of Fig 10 indicated that the VSOTFs varied regardless of the LCA and that the type of IOL did not affect the VSOTF given HOA. Ravikumar et al. [1] similarly reported that the impacts of LCA on visual performance were limited by the presence of monochromatic aberrations. For eyes with an average level of aberrations, the impact of LCA does not depend on differences in IOL type. Since the differences of BCVA among the IOLs were not significant in our experiments, the results also showed a small impact of LCA on visual acuity even when the spherical error and astigmatism of the eyes were corrected by spectacles.

When we included individual astigmatism after the surgeries in the calculations of VSOTF (the data were not shown), the astigmatism was more dominant than HOA for the VSOTF. And Artal et al. [16] reported that the CS was improved only by correcting both SA and LCA. Therefore, when eyes have small monochromatic aberrations, the eyes implanted with AMO IOLs that have relatively smaller chromatic dispersions may show high CSs. In this study, a slightly better regression curve with AMO IOLs than those of the others is shown in Fig 11. If, in the future, cataract surgery could be conducted without exacerbating ocular aberrations, or if optics that correct well for ocular aberrations that include HOA could be developed, the smallness of the chromatic dispersion of the IOL might prove more useful.

### The LCA of the Le Grand schematic eye with IOLs

To investigate our results of Fig 2, indicating that the LCA of the eye with Alcon and Hoya depended on the IOL powers, we calculated the LCA of the eye with IOLs using the Le Grand schematic eye [18] and paraxial ray tracing [34]. We replaced the crystalline lens data of the schematic eye with the IOL lens data (Table 3). Our measured chromatic dispersions of the IOLs (Table 5) were used for the calculations. The ACD values were set to the average value for the eyes with IOLs, 3.32 mm [36].

The LCA_840-561_ of four surfaces of the corneal and IOL, total eye optics, corneal total, and IOL total are shown in Table 7. As shown in the rightmost column of Table 7, the LCA of AMO’s IOLs was much smaller than the LCA of the IOLs of Alcon and HOYA. To compare the results shown in Fig 2, the slope of LCA to the IOL power was calculated; the slopes were 0.02 for Alcon, 0.02 for Hoya, and 0.003 for AMO. The slopes of the LCA measurements (Fig 2) were 0.017 for Alcon, 0.019 for Hoya, and 0.003 for AMO. The calculated slopes coincided well with the measured slopes even though the slope of the measured AMO LCA was not statistically significant.

**Table 7:** The LCA_840-561_ of each surface, total LCA_840-561_, corneal LCA_840-561_, and LCA_840-561_ of IOL were calculated for each kind of IOL with the paraxial approximation.

	Anterior cornea; Surface 1	Posterior cornea; Surface 2	Anterior IOL; Surface 3	Posterior IOL; Surface 4	Total; 1+2+3+4	Cornea; 1+2	IOL; 3+4
Alcon +10 D	0.61	−0.02	−0.07	0.26	0.78	0.59	0.20
Alcon +20 D	0.61	−0.02	0.03	0.37	0.99	0.59	0.40
Alcon +30 D	0.61	−0.02	0.14	0.45	1.19	0.59	0.60
HOYA +10 D	0.61	−0.02	−0.03	0.23	0.79	0.59	0.20
HOYA +20 D	0.61	−0.02	0.07	0.32	0.98	0.59	0.39
HOYA +30 D	0.61	−0.02	0.16	0.43	1.17	0.59	0.59
AMO +10 D	0.60	−0.02	0.00	0.03	0.62	0.58	0.03
AMO +20 D	0.60	−0.02	0.02	0.05	0.65	0.58	0.06
AMO +30 D	0.60	−0.02	0.04	0.05	0.68	0.58	0.09

For all IOLs, the calculated LCA was mathematically proportional to the powers of the IOLs. On the other hand, the measured LCA of +30 D IOL was smaller than that of +20 D IOL for AMO’s IOLs. We concluded that this result was caused by measurement error; and the smallness of LCA of AMO’s IOL for that difference of LCA in the paraxial approximation between +20 D and +30 D was no more than 0.03 D for AMO’s IOL (Table 7).

### Abbe’s number of crystalline lens

We calculated the LCA using paraxial ray tracing for both the Le Grand schematic eye [18] and the Navarro schematic eye [39]. The chromatic dispersion data of the schematic eyes are summarized by Atchison et al. [30]. The LCA_840-561_ of each surface, total LCA_840-561_, corneal LCA_840-561_, and LCA_840-561_ of the lenses for the Navarro schematic eye, the Le Grand schematic eye, and the modified Le Grand schematic eye are shown in Table 8; to fit the Le Grand schematic eye to our HSWA results for the phakic eye, the Abbe’s number of the crystalline lens, 50, had to be replaced by 45. The Le Grand and Navarro schematic eyes were based on the same LCA measurements of Polack [40], but there were some differences in their chromatic dispersions. In Atchison’s summary [30], the Abbe’s number of the crystalline lens of the Navarro schematic eye is 47.5, close to our replacement value, 45. We also calculated the LCA of the human eyes with IOLs, using paraxial ray tracing and the chromatic dispersions of the Navarro schematic eye. We compared that result with the measured LCA, as shown in Fig 13. The slope of the Deming regression was 0.94, and the regression line was shifted to the right in the graph; which means that the LCA was larger in the calculation with the Navarro schematic eye than it was in the measurements. So in this study we used the Le Grand schematic eye for the calculation and modeling work.

**Table 8:** The LCA_840-561_ of each surface, total LCA_840-561_, corneal LCA_840-561_, and LCA_840-561_ of the lens were calculated for the Navarro schematic eye [39], the Le Grand schematic eye [18], and the modified Le Grand schematic eye with the paraxial approximation. We changed the Abbe’s number of the lens from 50 to 45 for the Le Grand Schematic eye.

	Anterior cornea; Surface 1	Posterior cornea; Surface 2	Anterior lens; Surface 3	Posterior lens; Surface 4	Total; 1+2+3+4	Cornea; 1+2	Lens; 3+4
Navarro schematic eye	0.65	0.05	0.03	0.13	0.87	0.71	0.16
Le Grand schematic eye	0.61	−0.02	0.07	0.18	0.84	0.59	0.25
modified Le Grand schematic eye	0.61	−0.02	0.11	0.29	0.98	0.59	0.39

We believe that our adjustment of the Abbe’s number of the crystalline lens of the schematic eye needs more justification. Sivak et al. [41] investigated the chromatic dispersions of ocular media of various vertebrates and found that the chromatic dispersions of the cornea, aqueous humor, and vitreous humor were almost the same; the dispersions were close to that of water. In addition, although we tried to adjust the chromatic dispersion of the cornea as the LCA calculated with the paraxial approximation was matched to the LCA measured by HSWA, the results were not realistic. When the ocular components—cornea, aqueous humor, lens, and vitreous humor—are separated in the air, the cornea has a negative and smaller power than the other components, so that the change in LCA according to the change of Abbe’s number of the cornea is small. We also tried to adjust the chromatic dispersions of the aqueous humor or the vitreous humor. The Abbe’s number of the aqueous humor, 53, needed to be replaced by 37; the Abbe’s number of the vitreous humor, also 53, needed to be replaced by 78. Although the changes were smaller than the change required for the cornea, the results must have been wrong. The results of our modifying the Abbe’s number of the lens from 50 to 45 seemed reasonable given the values that Sivak and Mandelman reported for lens core and peripheral, 34 and 28, respectively. But there was a problem with those reported values. They investigated the chromatic dispersion of lenses extracted from a Toronto eye bank by using an Abbe refractometer or a Pulfrich refractometer; but these measurements could not be started until 24 hours after extraction. Although they used mineral oil to keep the lenses wet, the lenses may still have become denatured.

To change the power of the lens is the other way to fit the calculated LCA with the measured LCA. We changed the curvatures of the crystalline lens of the Le Grand schematic eye, keeping the ratio between the anterior and posterior curvatures and the Abbe’s number of the lens as it was originally. To fit the LCA with the calculation, we needed to change the anterior curvature from 10.2 to 6.522 mm and the posterior curvature from −6 to −3.837 mm. Then the power of the modified lens was +35.5 D. The resulting curvatures were unrealistically strong.

We can confirm only that the LCA results for the phakic eyes in the range between the visible and the infrared reported by Fernández et al. [14, 42] were the same as our LCA results. So their measured LCA was also larger than that predicted on the basis of the Le Grand and Navarro schematic eyes.

### Estimation of lens power and lens dispersion from LCA

In Fig 4, the fitting curve of the chromatic dispersion of the Alcon and Hoya IOLs almost coincides with that of the phakic eyes. Fig 13 shows that the calculated LCA based on the estimated chromatic dispersions of IOLs and of the cornea, aqueous humor, and vitreous humor of the Le Grand schematic eye model coincided with the measured LCA. This suggests that the Le Grand schematic eye data used in this calculation is valid. With our modification of the Abbe’s number of the crystalline lens, described in the previous section, the Le Grand eye model should represent the human eye. Given our analysis of the data presented in Fig 4 and Fig 13, the LCA of the IOLs must be close to the LCA of the crystalline lens of the human eye.

In Fig 2, the LCA with the IOL eyes and the power of the IOLs are in a linear relationship. Logically, we might expect to retain this linear relationship if we replaced the IOLs with human crystalline lenses. Because the inverse of the relationships between IOLs and crystalline lenses is valid in consequence of the linear relationship, we might also logically conclude that we could derive the crystalline lens power from the LCA measurements.

Unfortunately, because of the way the real eye components deviated from the components of the Le Grand schematic eye and because of the errors in our measurements, these logical assumptions proved to be unrealistic. But if and when the errors introduced by such factors can be substantially reduced, it will be possible to estimate the power of a crystalline lens by using an LCA measurement. It is very important to know the power of crystalline lens in the clinic, especially when performing cataract surgery.

## Conclusions

We measured the LCA using the HSWA with three wavelengths for the subjects implanted with acrylic yellow-colored IOLs. The LCA_840-561_ was 0.96 ± 0.06 for the phakic eyes; and 0.96 ± 0.11, 0.80 ± 0.06, 1.01 ± 0.09, and 0.66 ± 0.06 for the IOLs of Alcon, HOYA NY-60, HOYA XY-1, and AMO, respectively. AMO’s IOL showed smaller chromatic dispersions than those of the others. The LCA_840-561_ was positively correlated with the powers of the IOLs for Alcon and HOYA XY-1. The simulations we performed of MTF or VSOTF confirmed that the differences in chromatic dispersions for different kinds of IOLs have less impact on visual performance in the presence of some extent of monochromatic aberrations for a 4-mm pupil diameter. On the other hand, the small chromatic dispersion may dramatically enhance visual performance when the monochromatic aberration is small. We also defined a new Abbe’s number of lens for the Le Grand schematic eye so that it was well matched with the LCA of the phakic eyes; we replaced the original Abbe’s number, 50, with 45.
